# Primary mediastinum Ewing’s sarcoma with pleural effusion: A case report and literature review

**DOI:** 10.1515/biol-2022-0669

**Published:** 2023-08-01

**Authors:** Xuhong Li, Shan Qi, Taiwen Zhu, Ying Jiang, Wei Wang

**Affiliations:** Department of Respiratory and Critical Care Medicine, Zhongnan Hospital of Wuhan University, Wuhan 430071, China

**Keywords:** extraskeletal Ewing’s sarcoma, mediastinum Ewing’s sarcoma, pleural effusion

## Abstract

Ewing’s sarcoma (ES) is an aggressive cancer in young adults. Primary ES occurring in the chest with pleural effusion is even rarer. We report the case of a 15-year-old girl who presented with intermittent chest pain occurring for more than 2 months and cough and wheezing for 10 days. Radiological imaging showed a large soft tissue mass with multiple small vessel shadows near the left mediastinum and bloody pleural effusion in the left thorax. ES was diagnosed by positive immunostaining for CD99, FLI-1, and NKX2 combined with fluorescence *in situ* hybridization detection of the EWSR1 gene arrangement. With chemotherapy, lung computed tomography revealed that the tumor had become much smaller, and the fluid was absorbed. We report a case of extraskeletal Ewing’s sarcoma (EES) in the mediastinum with pleural effusion, which is unusual and challenging. EES is a highly malignant tumor with a poor prognosis. Early diagnosis and treatment can improve the survival rate of patients.

## Graphic abstract the timeline of the treatment

**Figure:**
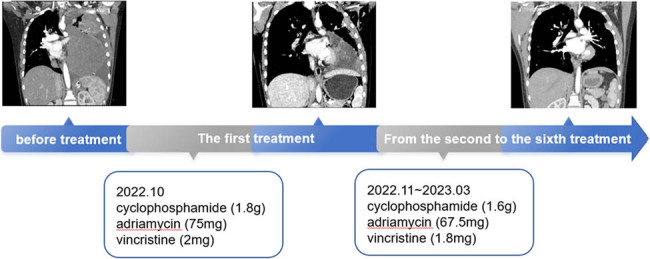

## 1 Background

Ewing’s sarcoma (ES) is a malignant tumor thought to originate from mesenchymal stem cells [1]. The tumor mainly occurs in children and adults, with a slight preference in males. It has an incidence rate of 4.5 per million per year in the population [2]. ES commonly occurs in the bones, especially the pelvis and limbs; however, it can also occur in many extraskeletal sites [3].

Extraskeletal Ewing’s sarcoma (EES) accounts for approximately 20% of ES cases and shows a bimodal age distribution among children and adults [4]. The incidence of EES is 0.4 per million individuals, and it has a high degree of malignancy and poor prognosis [5]. EES can occur in any soft tissue and is often misdiagnosed as other diseases. However, the most common sites are the upper thigh, buttocks, upper arm, and shoulders [4]. EES has also been reported to occur in the ribs, abdomen, and seminal vesicles [3,6,7]. However, EES originating from the mediastinum with massive pleural effusion has rarely been reported. Here, we report a case of EES with pleural effusion and review previous case reports to provide a better understanding of EES.

## 2 Case presentation

A 15-year-old girl was admitted to our hospital due to intermittent chest pain occurring for more than 2 months and cough and wheezing for 10 days. The patient was a student, and her family denied a history of malignancy. Chest examination revealed solid sounds obtained during percussion and low breath sounds over the left thorax.

**Figure 1:**
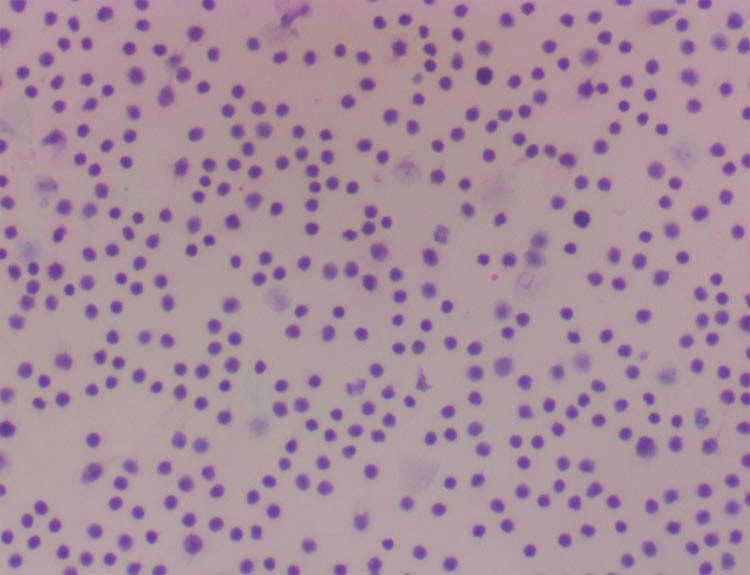
Examination of the pleural effusion showed massive lymphocytes, and no obvious heterotypic epithelial cells were detected.

The laboratory tests showed increased procalcitonin (26.59 ng/ml), elevated interleukin-6 (15.2 pg/ml), and a slightly high erythrocyte sedimentation rate of 28 mm/h. The patient underwent transcatheter thoracic drainage to determine the features of the pleural effusion, and a routine examination showed bloody turbid pleural effusion without tumor cells (Figure 1). However, the levels of carcinoembryonic antigen and lactic dehydrogenase in the pleural effusion were more than three times the normal upper limit. Acid-fast staining and tuberculosis gene sequencing of the pleural effusion were normal, helping us rule out the possibility of tuberculous pleural effusion.

**Figure 2:**
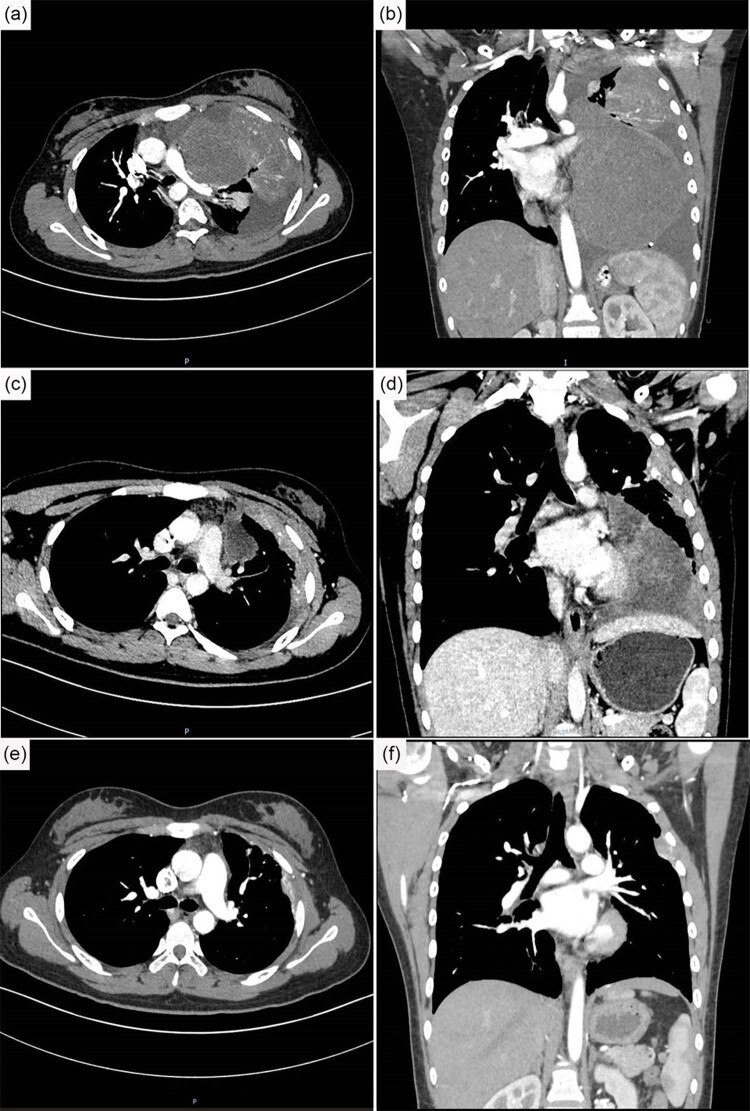
CT scan of the thorax. (a and b) A mass with multiple small vessel shadows was observed near the mediastinum. A pleural effusion was found in the left thorax. (c and d) Chest CT after one cycle of chemotherapy showed that the mass and fluid decreased. (e and f) After six treatment cycles, the tumor was hard to observe, and no pleural effusion could be observed. CT: computed tomography.

A chest computed tomography (CT) scan showed a large soft tissue mass (15.7 × 11.7 × 10.5 cm) near the left mediastinum with multiple nodules and masses in the left pleura, and the fluid could be seen on the same side of the thorax (Figure 2a and b). Moderately heterogeneous enhancement with multiple small vessel shadows could be seen in the aforementioned mass and nodules after the injection of a contrast agent (Figure 2a and b). A radiological examination of the abdomen and brain revealed no other lesions.

**Figure 3:**
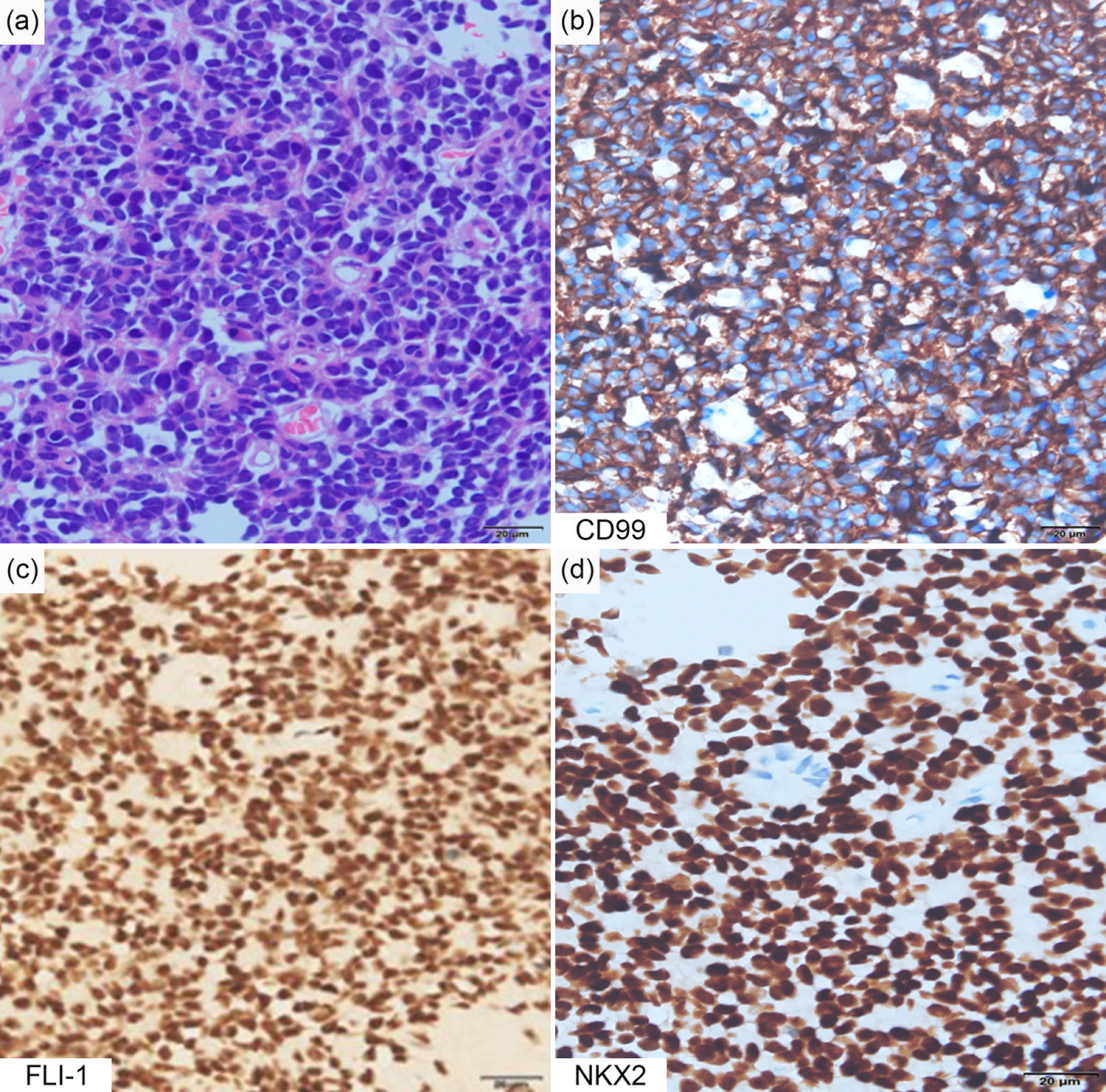
Pathological examination of the tumor. (a) Hematoxylin and eosin staining of the mass showed small and deeply stained cells with less extracellular matrix. (b–d) Immunohistochemistry of sections showed positivity for CD99, FLI-1, and NKX2. Scale bar = 20 μm.

**Figure 4:**
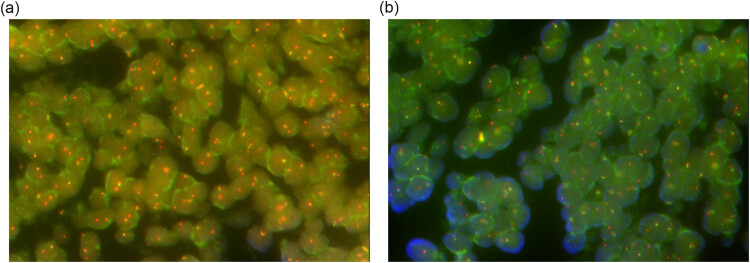
Molecular pathology of the tumor was detected by FISH. (a and b) Molecular pathology indicated EWSR1 rearrangement. The percentage of cells with EWSR1 breakage (red and green signal points separated) in the visual field was approximately 71%.

A percutaneous puncture biopsy was performed to clarify the properties of this mass. Pathological examination showed a population of small, round, deeply stained tumor cells. The tumor cells formed clusters. Immunohistochemical results demonstrated strong positivity for CD99, FLI-1, and NKX2, which are the markers of EES. The cells were also positive for Ki‐67 (approximately 30%). The tumor cells were negative for MPO, GFAP, CD30, EMA, CgA, desmin, and WT-1 (Figure 3). The negativity for these markers excluded lymphoma and glioma. After discussion by a multidisciplinary treatment (MDT) team, the mass was initially identified as ES derived from the mediastinum. Fluorescence *in situ* hybridization (FISH) suggested EWSR1 gene breakage and rearrangement (Figure 4). Based on the aforementioned results, the patient was diagnosed with left mediastinum ES with pleural effusion.

Considering the age, tumor size and properties, and the consensus from the MDT meeting, the patient received treatment with cyclophosphamide (1.8 g), adriamycin (doxorubicin) (75 mg), and vincristine (2 mg) (VDC) for the first cycle. After finishing one cycle of chemotherapy, a CT scan of the chest revealed that the tumor was approximately half its size before treatment, and the pleural effusion had decreased (Figure 2c and d). After approximately 3 weeks, the patient received cyclophosphamide (1.6 g), adriamycin (doxorubicin) (67.5 mg), and vincristine (1.8 mg) for the second cycle. After six cycles of treatment, the tumor was hard to observe, and no pleural effusion was observed (Figure 2e and f). The patient felt no chest pain and did not cough or wheeze. For the ongoing treatment plan, a regular chemotherapy will be continued, and the patient is advised to follow up.


**Informed consent:** Informed consent has been obtained from all individuals included in this study.
**Ethical approval:** The research related to human use has been complied with all the relevant national regulations, institutional policies, and in accordance with the tenets of the Helsinki Declaration, and has been approved by the authors’ institutional review board or equivalent committee.

## 3 Discussion

EES and ES both belong to the ES family of tumors, which has a common histology and genetic mechanism. ES is the second most common bone cancer in children, and EES only occurs in 20% of ES cases [8]. However, EES has a high degree of malignancy and a poor prognosis. Patients with EES often suffer from localized pain and present with nonspecific symptoms [8]. Thus, it may be mistaken for other diseases. Therefore, differential diagnosis is particularly important.

ES and EES are characterized by monomorphic, small, round blue cells. Small round cell sarcomas (SRCSs) share histological similarities with ES. Capicua transcriptional repressor (CIC)-rearranged sarcoma (CIC sarcoma) and BCOR-rearranged sarcoma (BCOR sarcoma) are two typical SRCSs [9]. CIC sarcoma is composed of slightly pleomorphic round cells [10]. However, BCOR sarcoma is composed of a mixed proliferation of round and spindle cells [11]. Immunohistochemically, CIC sarcoma and BCOR sarcoma are positive for CD99, which is an important marker of ES. CIC sarcoma can express patchy CD99, whereas CD99 is diffusely expressed in ES [11]. The biggest difference between SRCSs and ES is the gene mutation. CIC sarcoma often shows CIC–DUX4 fusion [12]. BCOR sarcoma often presents as BCOR–CCNB3 fusion [11]. However, ES is defined by fusions between FET and ETS, with approximately 85% of tumors expressing the EWSR1–FLI1 fusion [13].

Early diagnosis and treatment of EES can improve the survival rate of patients. The diagnosis is mainly based on imaging and histopathology. Imaging plays an important role in the evaluation and treatment management of EES [14]. CT findings of EES include a large mass with a similar intensity to that of the soft tissue. Calcification occurs in 10% of cases [4]. Although the imaging findings of CT are nonspecific, chest CT is important to assess the tumor size, shape, and boundary. Contrast-enhanced CT helps to assess the blood supply of the EES. Immunohistochemically, ES cells are positive for CD99, NKX2, and FLI-1 [15]. S-100 protein, CD57, neurofilaments, cytokeratin, and desmin are nonspecific markers of ES [11]. Additionally, the detection of EWSR1–FLI1 fusion is helpful in diagnosis. In our case, the patient exhibited cough and wheezing because of pleural effusion. Pleural effusion in patients, especially children or adolescents, is often mistaken for tuberculous hydrothorax, as tuberculosis is one of the main reasons for pleural effusion in our country. However, after further extraction of the pleural effusion, we found a large mediastinal mass by CT and performed a puncture biopsy. Immunohistochemistry of the mass showed positivity for CD99, FLI-1, and NKX2. Finally, the mass was diagnosed as EES after a discussion at the MDT meeting.

Treatment for EES is similar to that for ES. The current management of primary ES is a combination of cytotoxic drugs and local management, which includes surgery and radiotherapy [8]. Multiagent chemotherapy is critical to the treatment of ES. Vincristine, adriamycin (doxorubicin), and cyclophosphamide alternating with ifosfamide and etoposide (VDC/IE) are the standard treatments for ES in North America [16]. In Europe, vincristine, ifosfamide, doxorubicin, and etoposide are used for induction therapy. After induction therapy, high-dose chemotherapy with subsequent stem cell rescue can be used as consolidation treatment [16]. Definitive local therapy is recommended after induction chemotherapy [17]. For local control, surgery is performed in cases where complete resection is possible. Although ES is radiosensitive, the long-term outcome of radiotherapy alone for local control is less effective [4]. Radiotherapy could be used for local control when the mass is unresectable. In our case, the patient received chemotherapy and had an excellent outcome; therefore, she did not receive surgery and radiotherapy temporarily.

Although pulmonary EES has been reported in previous case reports, primary EES in the mediastinum with massive pleural effusion is rare [18]. We used “Sarcoma, Ewing’s, Ewing’s Sarcoma, Ewing’s Tumor” and “Effusion, Pleural, Pleural Effusions” as the keywords for a literature search and excluded publications regarding tumors originating from the chest wall. Here, we have summarized six cases from 2011 to 2023 [19,20,21,22,23,24] (Table 1). Of these six patients, four were female and two were male, exhibiting cough, dyspnea, chest pain, and tightness as the main clinical presentation. Among these patients, the oldest was 66 years of age, which is very rare. In addition, old age also contributes to poor outcomes. Of these cases, only one patient had gene examinations performed [20]. We consider gene examination combined with pathology to be more accurate. Three cases exhibited positivity for Ki-67, and we believe that this marker may be inversely correlated with the survival rate [19,21,24]. Primary EES in the mediastinum is easily mistaken for other tumors. Aldo Caltavituro et al. reported a 30-year-old female with recurrent EES in the mediastinum, which was mistaken for thymic carcinoma [24]. Because of the recurrent EES, they treated the patient with nine cycles of VDC/IE and two courses of high-dose chemotherapy followed by autologous transplantation. Our patient received different treatments and, similar to that 30-year-old female, has had an excellent outcome thus far. However, our patient still needs further long-term follow-up. Additionally, surgery was performed to relieve the symptoms in four cases [19,21,22,24]. This highlights the importance of local management.

**Table 1:** Primary EES with massive pleural effusion

First author	Publication date	Age (y)	Gender	Primary site	Clinical manifestation	Diagnosis method	Ki-67	Therapy	outcome
Aldo Caltavituro	2023	30	Female	Mediastinum	Cough, dyspnea	Pathology	40%	Surgery	Follow-up
								Chemotherapy (VDC/IE^a^, HDCT/ASCT)	
Manman Cui	2023	66	Male	Mediastinum	Chest tightness	Pathology	90%	Surgery	Death
Xuefeng Ling	2021	15	Female	Lobe	Chest pain, cough, dyspnea	Pathology	>70%	Surgery	Follow-up
								Chemotherapy (VDC/IE^a^)	
Xuexue Zou	2021	14	Male	Pleura	Fever, dyspnea	Pathology FISH	NS^b^	Chemotherapy	Death
								(VDC/IE^a^)	
								Radiotherapy	
Abhijith Bhaskaran	2021	34	Female	Pleura	Fever, cough, dyspnea	Pathology	NS^b^	Chemotherapy, surgery, radiotherapy	NS^b^
C Karatziou	2011	21	Female	Visceral pleura	Fever, cough, chest pain	Pathology	NS^b^	Chemotherapy	NS^b^

Abbreviations: VDC: vincristine, adriamycin (doxorubicin), cyclophosphamide; IE: ifosfamide, etoposide; HDCT/ASCT: high-dose chemotherapy followed by autologous stem cell transplantation. ^a^VDC/IE: vincristine, adriamycin, and cyclophosphamide alternating with ifosfamide and etoposide. ^b^NS: not stated.

In summary, EES is a highly aggressive tumor with a poor prognosis that can occur in many parts of the body. The diagnosis should involve a combination of clinical manifestations, CT imaging, and multidisciplinary discussion. Image-guided needle biopsies are especially important because they can provide early diagnosis. To improve the long-term survival rate of patients, chemotherapy should be adopted in a timely manner in combination with surgery or radiotherapy.

## 4 Conclusion

In this study, we report a rare case of primary mediastinum EES with massive pleural effusion. EES is a highly malignant tumor with a poor prognosis. Reports of this disease are rare, and there is little experience in treatment. The diagnosis and treatment of this disease require the joint discussion and analysis of experts from multiple disciplines (including respiratory, radiology, pathology, thoracic surgery, and oncology). Therefore, we must summarize the characteristics of more cases to provide evidence for further early diagnosis and improve the survival rate of patients.
